# Supplementary material for: Patterns of Intron Gain and Loss in Fungi
Source: PLoS Biol. 2004 Nov 30;2(12):e422. doi: 10.1371/journal.pbio.0020422 (PMC532390; doi:10.1371/journal.pbio.0020422)
Supplement: Table S1 — Also available at http://genes.mit.edu/NielsenEtAl/. (4.3 MB ZIP). [file pbio.0020422.st001.zip › NielsenEtAl/html/179.html]

AN6563.1.NCU03826.1.MG06936.1.FG07401.1


```
 CLUSTAL W (1.82) Multiple Sequence Alignments - Introns Inserted


Sequence 1: NCU03826.1	408 aa
Sequence 2: MG06936.1	412 aa
Sequence 3: FG07401.1	427 aa
Sequence 4: AN6563.1	411 aa
Alignment Length: 434 aa
Number Identitical Residues: 176 aa
Alignment Score (without introns) 9310


MG06936.1 	MSFGKLYTYE0KNPRSTAILAVAKANDTPLEVVNVDQ----EKNIPADYTSINKLGKVPT
NCU03826.1	MAFGKLYTYE0ANPRSTAILAVAKANNLDLEVIKVDL----EAAI-EEYKKVNPLGKVPT
FG07401.1 	MAFGTLFTRE0NNCRSTAIKAVAKANDIELNIVEAEK----GNAT-AEHLKANGLGKIPA
AN6563.1  	MAFGKLYGRP0DNTRTIAVLVAAKHNDLELELVETQANPAADFNKSDAYTKIQPLGKIPA
          	*:**.*:     * *: *: ..** *:  *::::.: ..::    .  : . : ***:*:

MG06936.1 	FVGADGYVLYECIAIAVY-~-V1TSQNEKTTLLGKTKQD2YASILKWMSFFNQEILPHMG
NCU03826.1	FVGADGYTLFECIAIAIY-~-V1ASQNEKTTLLGKTKQD2YASILKWLSFFNTEVLPPLA
FG07401.1 	FVGEDGFALSECIAIAIYN1AV~TSQNEKTTLLGKTKQD~YASILKWMSFFNSEVLPNLI
AN6563.1  	FEGANGFTLSEVIAIAVY-~-V1TSQNEKTTLLGKTKQD~YASILRWLSFANSELLVSFG
          	* * :*:.* * ****:*   * :*************** *****:*:** * *:*  : 

MG06936.1 	AWYRPLLGKDQYNKKSVEDASKRALKAISVVEEYLRDNTFLVGERITLADLFSVGIIARG
NCU03826.1	GWYRPLLGKAPYNKKAVEDAQATALKAISVAEAHLKNNTFLVGERITLADLFATGIIARG
FG07401.1 	AWFSPLKGDSPYNKKTVDEAIKATEKNFAVVEEYLLHNTFLVSERITLADLFAAAIATRG
AN6563.1  	SWFRPLLGLDPYNKKNVEDASKAALKKLGVLNTHLTANTYLVGERITLADLFTASLLTRA
          	.*: ** *   **** *::*   : * :.* : :*  **:**.*********:..: :*.

MG06936.1 	FEFFFDTKWRQEYPNVTRWYTTVYDQPIYSAVAPKLELLETPALTNTPPKKAEQPKKEA-
NCU03826.1	FEFFFDKAWREQYPNVTRWYTTVYNQPIYSAVAPPFALLDTPKLTNVAPKKAEAPKPAAP
FG07401.1 	FQYFFDKHWRAEHPAVTRWFDTVRSQPIFSEVAEKVELLETVTLTN-PPKKADQPKKEAK
AN6563.1  	FATVIDKKVRADYPAVTRWYQTIIDQSAFKAVVENPVLID-EAIKYTPPKKEEKPKKEA-
          	*  .:*.  * ::* ****: *: .*. :. *.    *::   :.  .*** : **  * 

MG06936.1 	---------KPAAAPAAAAE-----EDDEPKPQPKPKHPCDLLPKAAVPIDEWKRQYSNN
NCU03826.1	---------KPAAAPAAAAE-----EPAEA---PKPKHPLEALPRASFPLDEWKRQYSNV
FG07401.1 	KEAKKEAKKEAAPAPAAAAAPAEAPAADEAPAAPKAKHPLEALGRPSFPLDEWKRQYSNI
AN6563.1  	-------------APAAAAP-----AAEEDKPAPKPKHPLEALGKPTLILDDWKRTYSNE
          	             ******         *  . **.*** : * :.:. :*:*** *** 

MG06936.1 	-PVPQAMEWFWKNMDLTKDYSIWKVEYKYNNELGVPFMSCNLIG~GFEQRLEGSRKQLMG
NCU03826.1	-DTPEALKWFWENVPFT-EYSIWKVNYKYNDELTLTFMSNNLIG~GFNNRLEASRKYLFG
FG07401.1 	KDHNEAMKYFWDNFNFE-EWSIWKVDYKYNEELTLTFMSNNLIG~GFNNRLEGSRKYIFG
AN6563.1  	DTRSVAMPWFWQNYKPE-EYSLWKVNYKYDNELKLTFMANNLIG1GFHARLEASRKYLFG
          	.    *: :**.*     ::*:***:***::** :.**: **** **. ***.*** ::*

MG06936.1 	SASVYGDGHGSFIQGAFVIRGQEHEPVFNVAPDWESYTFTKLDPNNEEDRKYVRDEWEWE
NCU03826.1	CASVYGTNNDSVIQGAFVIRGDDWKPVFDVAPDYESYEFTKLDPQNPEDRAFVEAEWSWD
FG07401.1 	CAAVYGENNDSVIQGAFVIRGQEHVPAFDVAPDWESYEFVKLDPSKPEDRQFVEDAWGWE
AN6563.1  	CQGVYGENYACVNRGVFLVRGQEALPAFDVAPDYESYEFIKLDHTNEADRKYVEDIWAWD
          	. .*** .  .. :*.*::**::  *.*:****:*** * ***  :  ** :*.  * *:

MG06936.1 	RTVVKDGKEYPHYDGKVFK
NCU03826.1	KPALVNGKEYPHASGKVFK
FG07401.1 	KDITVNGKVYKLADGKVFK
AN6563.1  	TPVVVDGKELPNVDGHVFK
          	     :**     .*:***
```
